# Model validation for a knowledge and practices survey towards prevention of soil-transmitted helminth infections in rural villages in Indonesia

**DOI:** 10.1038/s41598-023-27781-3

**Published:** 2023-01-25

**Authors:** P. Lee, J. M. Kurscheid, B. Laksono, M. J. Park, A. C. A. Clements, C. Lowe, D. E. Stewart, D. J. Gray

**Affiliations:** 1 School of Medicine & Dentistry, Griffith University, Gold Coast, QLD Australia; 2 Department of Global Health, Research School of Population Health, Australian National University, Acton, ACT Australia; 3 Health Systems Support Unit, Swiss Centre for International Health, Swiss Tropical and Public Health Institute, 4051 Basel, Switzerland; 4 Faculty of Medicine, Universitas Diponegoro, Semarang, 50275 Indonesia; 5 Department of Nursing, College of Nursing, Konyang University, Daejeon, South Korea; 6 Faculty of Health Sciences, Curtin University, Perth, Australia; 7 Department of Medical Research, China Medical University Hospital, China Medical University, Taichung, Taiwan

**Keywords:** Risk factors, Infectious diseases

## Abstract

The rate of soil-transmitted helminth (STH) infection is estimated to be around 20% in Indonesia. Health promotion and health education are cost-effective strategies to supplement STH prevention and control programs. Existing studies suggest that quantitative tools for knowledge, attitudes and practices (KAP) are important to monitor effective community-based STH interventions. However, evidence is limited regarding the applicability of such tools. This study aims to identify the socio-demographic predictors for STH-related knowledge and practices and validate the quantitative tools in population use. A cross-sectional study design was conducted among residents of 16 villages in Central Java, Indonesia. Adult and child respondents were interviewed to assess general knowledge and practices in relation to STH. Two mixed effects models identified the significant factors in predicting knowledge and practice scores. The model predicted knowledge and practice scores were compared with the observed scores to validate the quantitative measurements developed in this study. Participants’ socio-demographic variables were significant in predicting an individual’s STH-related knowledge level and their hand washing and hygiene practices, taking into account household-level variability. Model validation results confirmed that the quantitative measurement tools were suitable for assessing STH associated knowledge and behaviour. The questionnaire developed in this study can be used to support school- and community-based health education interventions to maximize the effect of STH prevention and control programs.

## Introduction

Soil-transmitted helminth (STH) infections, considered as neglected tropical diseases, remain one of the most common global public health issues in developing countries. It is estimated that 1.5 billion people (20% of the global population^1^) are infected with STH worldwide, with the poorest and most deprived communities typically affected^2^. The main STH species that infect human populations include *Ascaris lumbricoides* (roundworm), *Trichuris trichiura* (whipworm) and hookworm (the two species). These parasites are transmitted by eggs shed in human faeces which contaminate the environment in areas with poor hygiene and sanitation.

In Indonesia, one-fifth or more of the population is estimated to be infected with *A. lumbricoides* or hookworms and approximately 12% infected with *T. trichuria*^3^.

The World Health Organization (WHO) recommends periodic mass drug administration (MDA) targeting at-risk populations such as school-aged children and adults in STH endemic areas^2,4,5^. Administration of anthelmintics such as albendazole is considered a safe and effective control strategy to tackle STH infections at a community level. Supplemental prevention strategies are often encouraged in addition to periodic deworming, including improvements in environmental sanitation, provision of clean water supplies, promoting latrine use, access to health services and provision of health education. Such initiatives have been encouraged as important components of efforts to ensure sustainable and effective STH control programs^6–10^.

Health promotion and health education focusing on STH have been found to be cost-effective and affordable measures for disadvantaged communities, in combination with other intervention strategies. Health education programs can be designed to reduce contamination of soil and to promote the use of latrines and individual hygienic behaviour, thereby helping to prevent transmission and re-infection by STH^11–14^.

However, there is an urgent need for systematic and validated measurement tools to measure knowledge, attitudes and practices (KAP) related to STH. Relevant studies typically design a knowledge questionnaire including the following aspects: signs and symptoms, transmission of infection and perceived causes, treatment and prevention measures with respect to STH infections^8,15–17^. Measurement tools for practices or behaviour associated with STH prevention vary and the included contents are often inconsistent. These usually include questions about personal hygiene practices such as hand washing, washing fruits and vegetables, boiling water for drinking, wearing shoes and participant history of deworming, but often lack standardized scales^8,17,18^. We have observed that recent studies present simple frequency distributions of responses to all sub-questions under each KAP section. Only a few studies conduct univariate analyses (in relation to socio-demographic characteristics) and assess associations between knowledge or practices with STH outcomes^8,16,19^. Evidence is limited in determining the relationship between knowledge and practices, as well as their influence on STH outcomes. There is also a lack of established quantitative tools to assess STH-related knowledge and practices in large populations. The objective of this study was therefore to identify the socio-demographic predictors for knowledge and practices related to STH and validate the quantitative tools in population use.

## Methods

### Study design and data collection

A cross-sectional study design was employed to collect survey data from residents of 16 villages involved in an overarching sanitation improvement study in Central Java, Indonesia, during February-April 2014. As reported in a recent publication, participants were recruited through random selection of households in the villages. Face-to-face interviews were carried out by trained research assistants, consisting of local nurses, midwives and public health workers^20^.

Two structured questionnaires (for adult and child) were developed in English and translated to Bahasa Indonesia (Appendix A and B). The adult questionnaire (for participants aged 13 years and above) elicited details on demographics, latrine use, water access and usage, hand washing practices, knowledge and behaviour associated with STH, together with a section for interviewers to observe and record the condition of respondent’s hands and nails (i.e. nail biting and cleanliness). The child questionnaire (for respondents aged 2–12 years) was identical, with the exception that the sections on housing conditions and animals were omitted. Children were also not asked questions on water usage beyond the source of water for drinking. Parents of children below the age of five were asked to answer on behalf of their child^20^.

### Measurements of knowledge and practices

STH knowledge and behaviour (hand washing and hygiene practices) were measured using two scales containing 18 and 10 questions respectively and the total scores were calculated based on the number of correct responses to those questions. Further details of the knowledge and practice scales have been presented elsewhere^20^.

### STH infection status

Stool samples were collected from all children and adult respondents. Faecal sample collection and processing are detailed in the Research Team’s previously published article^20^. Prevalence of each type of STH infection is determined based on the percentage of individuals receiving positive test results out of the total respondents in each cohort group. The outcomes included *Ascaris*, *Trichuris*, hookworms and any STH infection (the presence of at least one of the previous three types of infections).

### Data analysis

Data analysis was performed using SPSS Statistics 24. Descriptive statistics including frequency distribution, mean and standard deviation (SD) were calculated for participant demographic characteristics and each of the outcome variables, knowledge (about STH) and behaviour (hygiene and sanitation practices in preventing STH) scores. In order to make better sense of the comparisons of knowledge and behaviour scores between subgroups within the study samples, the total scores were converted to percentages of correct responses in the scales [(total correct responses/total number of question) × 100%] for each participant. Independent sample t-test (or Mann–Whitney U test as the non-parametric alternative) and One-Way ANOVA (or Wilcoxon signed Rank test) were carried out to assess the mean differences in the outcome scores between different subgroups (different categories under each demographic variable, e.g. male and female subgroups under ‘gender’). In addition, Spearman’s correlation analyses were performed to examine the relationships between age, knowledge and practice scores. Due to the hierarchical nature of the data, the measurements taken from members within the same household are likely to be correlated (they are not independent) but different to those of participants from other households. Thus, two mixed effects models were performed to identify the significant factors in predicting knowledge and behaviour scores in consideration of individual and household characteristics in the adult and child combined samples. Histogram distributions, scatterplots and Pearson’s correlations were applied to compare the observed and predicted values of knowledge and behavior scores for model validation. Statistical significance was set at p < 0.05.

### Ethics

Ethical approval for this study was obtained beforehand from the Semarang City authorities (ref. 070/613/IV/2011), and from the Human Research Ethics Committees at Diponegoro University and at Griffith University (ref. PBH/17/11/HREC). Methods were carried out in accordance with relevant guidelines and regulations and informed consent was obtained from all subjects and/or their legal guardians(s).

## Results

Table 1 displays the socio-demographic characteristics of respondents. A total of 6,466 individuals (from 2195 households) responded to the survey, of which 82% were adults (including adolescents, n = 5303) and 18% were children aged 12 and younger (n = 1161). Of the study participants, 50.2% (n = 3229) were males. The child sample cohort had a slightly higher proportion of males than the adult sample cohort (52.5% vs. 49.7%). The age rage of the participants were from 2 to 93 and the mean ages were 39.3 (± 17.2) and 7.0 (± 3.0) between the two samples, respectively.

**Table 1:** Demographic characteristics of respondents.

Variable	Total (N = 6466)	Adult (N = 5305)	Child (N = 1161)
	No. (%)	No. (%)	No. (%)
**Gender**			
Male	3229 (50.2)	2619 (49.7)	610 (52.5)
Female	3205 (49.8)	2654 (50.3)	551 (47.5)
**Age (years)**			
Mean (SD)	33.5 (19.9)	39.3 (17.2)	7.0 (3.0)
**Education**			
Not at school (< 6 y/o)	521 (8.3)	–	521 (45.3)
Grades 1–6 (Elementary school)	1374 (21.9)	744 (14.5)	630 (54.7)
Grades 7–9 (Junior school)	2041 (32.5)	2041 (39.9)	–
Grades 10–12 (Senior school)	1261 (20.1)	1261 (24.6)	–
College or higher	1076 (17.2)	1076 (21.0)	–
**Occupation**			
Child not at school	521 (8.2)	–	521 (45.3)
Student	1193 (18.7)	582 (11.1)	630 (54.7)
Private sector	494 (7.7)	494 (9.4)	–
Farmer	346 (5.4)	346 (6.6)	–
Public sector	54 (0.8)	54 (1.0)	–
Self-employed	586 (9.2)	586 (11.2)	–
Unemployed	595 (9.3)	595 (11.3)	–
Home duties	869 (13.6)	869 (16.6)	–
Other/unspecified	1720 (27.0)	1720 (32.8)	–
**Income bracket (IDR**)			
Low (below 700 K)	1229 (24.6)	1229 (24.6)	N/A
Low-mid (700–999 K)	757 (15.1)	757 (15.1)	
Upper-mid (1–1.9 M)	1635 (32.7)	1635 (32.7)	
Higher (≥ 2 M)	1382 (27.6)	1382 (27.6)	

The majority of the respondents had attained education up to the end of junior school (54.4%of the overall respondents) and 17.2% of them were considered well educated with college and higher levels of education. As the child sample in this study consisted of children aged 12 and younger, the highest level of education in this sample was elementary education (54.7%). Of the 1161 children, 35.9% was aged 5–11. In the children sample, 0.2% in the younger sub-group (aged 5–11) and 85.2% in the older sub-group (aged 6–12) were at school at the time of interview (data not shown). The employed adults worked in various occupation categories such as self-employed (11.3%), private sector (9.4%), farmers (6.6%) and public sector (1.0%). Almost one third of the adults indicated ‘other or unspecified’ category to describe their occupations. In addition, over 70% of the responding adults were students (11.1%), doing home duties (16.6%) self-employed (11.3%) or classified as other or unspecified occupation (32.8%). In terms of household income, only adult participants responded to this question. Over 60% (n = 3017) of the study participants reported a monthly household income of ≥ 1 M IDR (equivalent to approximately $2.77 USD based on the average 2014 exchange rate).

### Knowledge of STH (knowledge score) and demographic factors

The comparisons of participants’ levels of knowledge of STH across different socio-demographic factors are outlined in the first part of Table 2. Adults had a significantly higher mean knowledge than children (57.3% vs. 42.9%, p < 0.001). Overall, a weak positive association between knowledge score and age was observed (rho: 0.16, p < 0.001). However, the results also indicated a strong positive correlation between knowledge score and age in the child cohort (rho: 0.51, p < 0.001) but a negative association in the adult cohort (rho: − 0.22, p < 0.001).

**Table 2:** Knowledge and behaviour scores by demographic factors.

Demographic variable	Knowledge score (%)			Behaviour score (%)		
	Mean	SD	p-value	Mean	SD	p-value
**Child/Adult**^a^						
Child	42.9	18.0	< 0.001	61.2	20.4	< 0.001
Adult	57.3	10.4		69.0	13.1	
**Age** (correlation)^b^	rho: 0.16		< 0.001	rho: 0.12		< 0.001
Age (Child)	rho: 0.51		< 0.001	rho: 0.35		< 0.001
Age (Adult)	rho: − 0.22		< 0.001	rho: − 0.03		0.038
**Gender**^c^						
Female	55.0	13.3	0.12	69.0	14.7	< 0.001
Male	54.5	13.4		66.2	15.1	
**Education**^d^						
Not at school	30.9	16.7	< 0.001	54.1	22.3	< 0.001
Grades 1–6	51.9	13.2		67.1	15.0	
Grades 7–9	56.3	10.1		68.6	13.2	
Grades 10–12	59.1	9.0		69.7	12.7	
College or higher	59.9	8.6		69.7	12.8	
**Occupation**^e^						
Not at school (Child)	30.9	16.7	< 0.001	50.2	24.2	< 0.001
Student	52.2	15.3		66.5	15.7	
Home duties	57.2	9.8		70.4	12.9	
Public sector	65.1	6.8		78.2	10.6	
Farmer	54.5	11.0		69.0	12.8	
Private sector	60.5	8.2		69.3	11.6	
Self-employed	58.7	9.2		67.4	12.4	
Unemployed	54.3	12.7		68.0	14.6	
Other	56.7	10.4		69.1	13.0	
**Income bracket**^f^						
Low (below 700 K)	55.0	11.6	< 0.001	69.2	13.4	< 0.001
Low-mid (700–999 K)	56.4	11.4		69.5	13.4	
Upper-mid (1–1.9 M)	58.3	9.0		70.2	12.9	
Higher (≥ 2 M)	59.2	8.9		67.7	12.9	
**Knowledge score (%)**^g^	NA		NA	rho: 0.48		< 0.001

^a,c^Independent sample t tests were performed.

^b,g^Spearman correlation tests were performed.

^d,e^One-Way ANOVA tests were performed.

^f^Wilcoxon test was performed.

**Figure 1:**
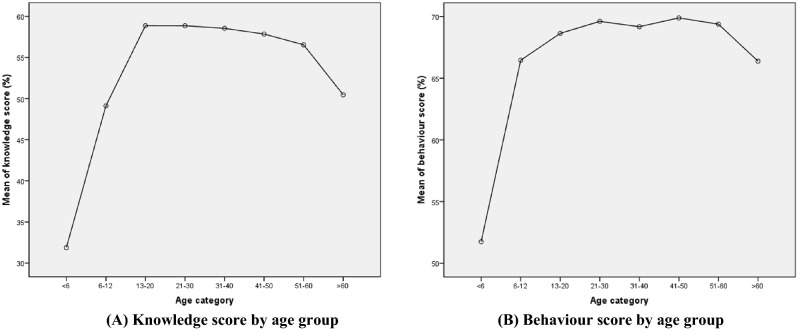
Mean scores of observed knowledge and behavior scores (%) by age group.

Figure 1A depicts the pattern of change in knowledge score by age group. A steep increase was observed among children and a stable but gradually decreasing trend with age was found in adults (except a sharp decrease from aged > 60). Significant differences in mean knowledge scores were also identified between education, occupation and income groups (p < 0.001 in One-Way ANOVA with post-hoc tests, Table 2). Knowledge scores increase with education levels. The mean knowledge score among adults with college, or above, levels of education was almost twice that of the score for children not at school (mean scores: 59.9 and 31.9 respectively). The post-hoc tests indicated a significant difference between any pair of the education groups, except the two highest levels of education (Grades 10–12 and college and higher, p = 0.53, data not shown). Knowledge scores were lowest among students, farmers and unemployed participants and highest among public sector employees.

### Hand washing and hygiene practices (behaviour score) and demographic factors

The comparisons of participants’ behavior scores measuring their hygiene and sanitation practices with regard to STH (abbreviated as behaviour score) across different socio-demographic factors are presented in the second part of Table 2. Adult participants had a significantly higher mean behaviour score than children (69.0% vs. 61.2%, p < 0.001). Behaviour scores were positively associated with age in the whole sample (rho: 0.12 p < 0.001). Further analysis revealed that a positive association was found between age and behaviour score in children but a weak negative association was identified in adults (rho: 0.35 and − 0.03, p < 0.001 and p = 0.038 respectively). Figure 1B indicated that the mean behaviour score in children aged < 6 was significantly lower than those of the other age groups (51.7 vs. score range: 66–70). Behaviour score (in Table 2) increased with education (p < 0.001, with no difference between any pair of the three highest education levels in post-hoc comparisons, p > 0.05, data not shown). Significant differences in mean behaviour scores were also identified between occupation and income groups (p < 0.001 in One-Way ANOVA tests). The results of post-hoc analyses showed that public sector employees practiced greater levels of hygiene compared to other occupation categories, whilst students, self-employed and unemployed participants practiced the lowest levels (excluding children not at school).

### STH outcomes and mean differences in knowledge and behaviour scores between infected and uninfected individuals

Table 3 presents the results of STH prevalence and the differences in knowledge and behavior (practice) scores between infected and uninfected participants in the child and adult samples. The overall STH infection (infected with at least one type of STH) rates were 32.7% (n = 380) in children and 34.1% (n = 1810) in adults. The results also showed that children without any STH infections had consistently higher knowledge and practice scores than those infected with different types of STHs. Uninfected children showed a significantly higher knowledge score than those infected with at least one type of STH (43.74 vs. 41.24, p = 0.025) and than those with *Ascaris* infections (43.62 vs. 41.12, p = 0.034). In the adult sample, uninfected participants show a much higher level of hand washing and hygiene practices than those infected with Trichuris (69.11 vs 64.08, p < 0.001).

**Table 3:** Results of STH outcomes and comparisons of knowledge and practice scores between infected and uninfected groups.

	Children (N = 1161)			Adults (N = 5305)		
	Uninfected	Infected	p value	Uninfected	Infected	p value
**Any STH infection** N(%)	781 (67.3)	380 (32.7)		3495 (65.9)	1810 (34.1)	
Knowledge M(SD)	43.74 (18.12)	41.24 (17.62)	0.025*	57.18 (10.41)	57.57 (10.43)	0.198
Practice M(SD)	61.87 (20.57)	59.74 (20.14)	0.096	68.87 (12.95)	69.30 (13.30)	0.260
**Ascaris** N (%)	839 (72.3)	322 (27.7)		3945 (74.4)	1360 (25.6)	
Knowledge M(SD)	43.62 (18.13)	41.12 (17.52)	0.034*	57.16 (10.52)	57.78 (10.13)	0.058
Practice M(SD)	61.57 (20.73)	60.14 (19.69)	0.287	68.83 (12.99)	69.57 (13.28)	0.072
**Trichuris** N (%)	1141 (98.3)	20 (1.7)		5209 (98.2)	96 (1.8)	
Knowledge M(SD)	42.98 (17.96)	39.60 (19.85)	0.405	57.33 (10.40)	56.67 (11.53)	0.538
Practice M(SD)	61.31 (20.36)	53.54 (24.11)	0.092	69.11 (13.07)	64.08 (11.87)	< 0.001***
**Hookworms** N (%)	1103 (95.0)	58 (5.0)		4852 (91.5)	453 (8.5)	
Knowledge M(SD)	42.93 (17.99)	42.83 (18.08)	0.966	57.39 (10.27)	56.51 (11.86)	0.128
Practice M(SD)	61.32 (20.34)	58.37 (22.40)	0.284	68.97 (13.04)	69.50 (13.380	0.409

**Notes:** 1. Mann–Whitney U tests were performed to compare the differences in knowledge and practice scores (in percentage between uninfected and infected groups; 2. Significance levels: *p < 0.05, **p < 0.01, ***p < 0.001.

### Results of mixed effects model in predicting knowledge scores

The mixed effects model approach takes into account the variables at both individual (fixed effects) and household levels (random effects) to predict the changes in knowledge and behavior scores. In the process of finding the best fitting model for knowledge score, various combinations of multiple demographic factors were included in different models to compare the model performances with the null model (including household only) [− 2Restricted Log Likelihood (− 2LL), Akaike’s Information Criterion (AIC) and Schwarz’s Bayesian Criterion (BIC) as the indicators for model comparison]. Due to strong correlations, education, occupation and income status were included independently in different models together with gender, adult/child and age to avoid multi-collinearity among these three variables. In order to consider the very different patterns in the change of knowledge scores with age between adult and child samples, an interaction term between adult/child and age (age* adult/child) was added into the model (Table 4). All the model assessment indicators show the best fitting outcome when education was included with the first three demographic variables (gender, adult/child and age) in the model. The final model including education performed slightly better than the model including occupation (htly better than the m2LL, AIC and BIC values: 46,656–46,675 and 47,347–47,364) and improved significantly compared with the null model (htly better than the m2LL, AIC and BIC values: 51,259–51,276; data not shown).

**Table 4:** Estimates of fixed and random effects for knowledge score (percentage).

(A) Estimates of fixed effects							
Parameter	Estimate	Std. Error	df	t	p value	95%CI	
						Lower	Upper
Intercept	61.49	0.46	6225.0	134.08	< 0.001	60.60	62.39
Gender (Male)^a^	− 0.57	0.23	4790.4	− 2.49	0.013	− 1.02	− 0.12
Adult/Child (Child)^b^	− 29.42	1.73	5108.8	− 17.04	< 0.001	− 32.80	− 26.03
**Education [College or higher]**^**c**^							
Not at school	− 8.55	1.14	5237.7	− 7.47	< 0.001	− 10.79	− 6.31
Grades 1–6	− 4.51	0.57	5894.7	− 7.92	< 0.001	− 5.62	− 3.39
Grades 7–9	− 1.93	0.40	5481.0	− 4.88	< 0.001	− 2.71	− 1.16
Grades 10–12	− 0.30	0.40	5298.9	− 0.74	0.458	− 1.08	0.49
Age	− 0.07	0.01	5671.5	− 6.70	< 0.001	− 0.09	− 0.05
Age*Adult/Child							
(Age*Child)^d^	2.54	0.17	4930.6	15.32	< 0.001	2.22	2.87

(B) Estimates of covariance parameters							
Parameter	Estimate	Std. Error	Wald Z	p value	95%CI		
					Lower	Upper	
Residual	69.63	1.54	45.14	< 0.001	66.67	72.72	
Intercept (Subject = Household) Variance	56.49	2.66	21.24	< 0.001	51.51	61.95	

^a^Reference group: Females.

^b^Reference group: Adults.

^c^Reference group: College.

^d^Reference group: Age*Adult.

In the final model, the results of ANOVA tests for the fixed effects of the included demographic variables show that the intercept and all the included demographic variables made significant contributions to the model (p value for gender: 0.013, all the other p values < 0.001) (data not shown). Table 4A summarizes the test results and parameter estimates for the fixed effects in the model. The baseline intercept indicates that the mean knowledge score percentage for youngest female adults with college level of education was 61.49 (using reference categories for the included variables, 95%CI: 60.60–62.39). Male respondents have a slightly lower score than females (mean score reduced by 0.57, p = 0.013). Being a child would have a significantly reduced mean score by 29.42 points (p < 0.001) than adults. In general, knowledge score increases with education levels. For example, the scores for participants who were not at school, had education levels between grades 1–6 and grades 7–9 were significantly lower than that of participants with college and above levels (p values < 0.001). However, the knowledge score among respondents with education between grades 10–12 was not significantly lower than those with college level education (p = 0.458). Age and (age*adult/child) were also significant (both p values < 0.001) in predicting knowledge score. The covariance parameters in Table 4B suggest that both residual (at the individual level) and intercept (at the household level) variance components are significant (both p < 0.001), meaning that the average knowledge scores vary among participants and also between households. About 44.8% [56.49/(69.63 + 56.49) = 44.79%)] of the total variance could be attributed to the differences in knowledge score between households.

### Results of mixed effects model in predicting behaviour scores

Similar to the process of model development for knowledge score, a series of model testing and assessment were applied to identify the best fitting model to predict participants’ behavior scores. In addition to the demographic factors and age* adult/child included in the previous model (Table 5), knowledge score was also added in the model considering its strong correlation with behavior score (rho: 0.48, p < 0.001). Along with household variance, practice score was also entered to test for random effects in explaining behavior score in the final model. The model assessment criteria (htly better than the m2LL, AIC and BIC) reduced from 51,461–51,419 for the null model to around 47,609–47,626 for the final model (htly better than the m-2LL, AIC and BIC data not shown), suggesting an improvement in model fitting. The results of ANOVA tests for the fixed effects of the intercept and all the included variables contributed significantly in explaining behaviour score (all p values ≤ 0.001) except education (p = 0.67, data not shown). The intercept of 43.36 indicates the mean behaviour score at baseline (young adult females and reference for all included variables). Being a male and a child would have a reduced mean score by 2.26 and 6.0 points than females and adults respectively (both p < 0.001). Both Age and (age* adult/child) were significant in predicting practice score (p values 0.002 and < 0.001 respectively). Knowledge score is also a significant predictor (p < 0.001). Every point increase in knowledge score increases behaviour score by 0.4. The covariance parameters in Table 5B show that both residual (individual characteristics) and intercept (household variations) covariances are significant (both p < 0.001), which indicated that behavior scores vary between households and among respondents. Between-households variability explained a large proportion (97.15/(71.27 + 97.15) = 57.68%) of the overall variance in practice score.

**Table 5:** Estimates of fixed and random effects for behaviour score (percentage).

(A) Estimates of fixed effects							
Parameter	Estimate	Std. Error	df	t	p value	95%CI	
						Lower	Upper
Intercept	43.36	0.97	6106.3	44.80	< 0.001	41.46	45.26
Gender (Male)^a^	− 2.26	0.24	4640.0	− 9.59	< 0.001	− 2.73	− 1.80
Adult/Child (Child)^b^	− 6.00	1.83	4946.7	− 3.28	0.001	− 9.58	− 2.42
**Education [College or higher]**^**c**^							
Not at school	− 1.19	1.19	4963.6	− 1.01	0.315	− 3.53	1.14
Grades 1–6	− 0.24	0.60	5562.9	− 0.40	0.688	− 1.42	0.94
Grades 7–9	− 0.24	0.41	5135.6	− 0.59	0.553	− 1.05	0.56
Grades 10–12	− 0.51	0.41	4996.2	− 1.23	0.218	− 1.32	0.30
Age	0.03	0.01	5363.1	3.05	0.002	0.01	0.05
Knowledge score (%)	0.45	0.01	6071.4	33.17	< 0.001	0.42	0.48
Age*Adult/Child							
(Age*Child)^d^	0.89	0.17	4768.1	5.15	< 0.001	0.55	1.24

(B) Estimates of covariance parameters							
Parameter	Estimate	Std. Error	Wald Z	P value	95%CI		
					Lower	Upper	
Residual	71.27	1.57	43.31	< 0.001	68.25	74.42	
Intercept (Subject = Household) Variance	97.15	3.88	25.04	< 0.001	89.84	105.06	

^a^Reference group: Females.

^b^Reference group: Adults.

^c^Reference group: College.

^d^Reference group: Age*Adult.

### Validation of the developed models in predicting knowledge and behaviour scores

**Figure 2:**
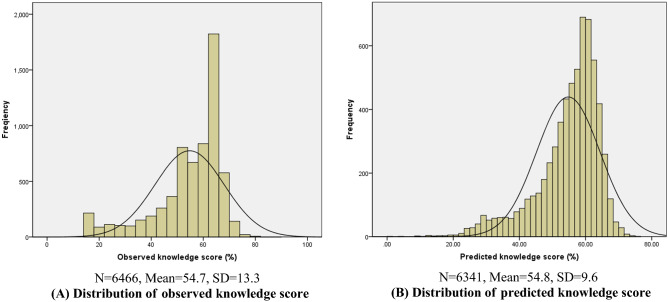
Distribution of observed and predicted knowledge scores (%).

The two final mixed effects models were used to compute predicted knowledge and behaviour scores for each participant. The predicted scores were compared with the knowledge and behaviour scores reported by the participants (observed scores). Figure 2 illustrates the comparison of observed and predicted frequency distributions of knowledge scores. The observed distribution has a distinct peak at the score 64 (mode value in the sample), while the predicted distribution peaking around 58–60, both slightly left skewed with mean values at 54.7 and 54.5, respectively. The comparison of quartile values (Supplementary Table 1A) show that the predicted scores tend to underestimate the observed scores (median values: 56.92 vs. 60). Spearman correlation analysis was used to assess how closely the overall predicted knowledge scores are to the observed scores at the individual level. The correlation coefficient of nearly 85% (see Supplementary Fig. 1, rho: 0.847, p < 0.001) indicates a strong correlation between the observed and predicted knowledge scores, suggesting a well-fitted model for the sample data.

**Figure 3:**
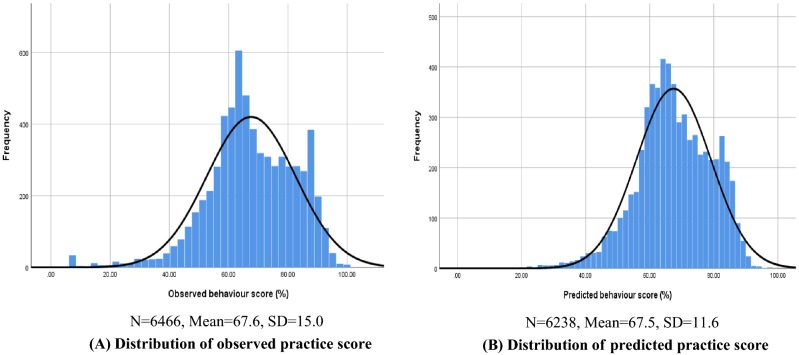
Distribution of observed and predicted behaviour scores (%).

The results from the final model, in consideration of the positive relationship between knowledge and behaviour scores, have confirmed that knowledge score was a significant predictor for behaviour score. Figure 3 presents the comparison between observed frequency distribution and predicted frequency distribution of knowledge scores calculated based on the developed mixed effects model. Overall, the predicted distribution was reasonably similar to the pattern of the observed distribution, with very accurate sample mean prediction (67.5 vs 67.6). Despite the minor under- or overestimates in predicting the quartile values (difference ranging from – 1.78 to 1.83, in Supplementary Table 1B), the result of Spearman correlation further confirmed well matched predicted and observed scores (Supplementary Fig. 2, rho: 0.883, p < 0.001). The validation results indicate that the final model fits the sample data (including adult and child sub-samples) and can be used to predict behaviour scores among the study respondents.

## Discussion

The findings of this study revealed that the participants’ socio-demographic variables such as age, gender and education in conjunction with household variability are significant in predicting individual’s knowledge level of STH. In addition, participant’s knowledge score coupled with their socio-demographic characteristics are significant predictors for their hand washing and hygiene practices, taking into account household-level variability. The findings are in line with previous studies which identified age, gender and educational level as the common associated demographic factors^8,19,21,22^. However, none of these studies combined adults and children in their analyses and examined the effect of household-level variation on respondents’ levels of knowledge and practices in relation to STH infections and prevention. Further, most of these studies assessed associations between socio-demographic characteristics and each single component/item (such as a specific transmission route or symptom) in their KAP questionnaires. Only one study^19^ used a summated scale (a combination of several questions) to measure general knowledge and explores its associations with children’s gender and age. The present study provided additional information to examine how knowledge interacted with participants’ socio-demographic characteristics (including being a child or an adult) to predict their washing and hygiene practices.

About one third of children and adults in the study communities had parasitic worm infections (32.7% and 34.1% respectively), and *Ascaris* was identified as the predominant species (27.7% in children and 25.6% in adults). The study also suggests that children without STH infections had consistently higher knowledge and practice scores than those with different types of STH infections, despite some statistically insignificant results. This finding is in agreement with a study showing lower hookworm prevalence among schoolchildren with better hygiene knowledge than those who lacked such knowledge in rural Cote d’Ivoire^19^. However, the insignificant differences in knowledge and practice scores between groups in terms of infection status with *Trichuris* (knowledge scores in uninfected group vs. infected group: 42.98 and 39.60; practice scores: 61.31 and 53.54., respectively), as well as level of practice in relation to hookworm infection (uninfected: 61.32 vs. infected: 58.37) could be due to small number of infected individuals (n = 20, 96 and 58 respectively) in the infected group for these analyses. The findings support the potential benefits of health education or STH prevention programs targeting children to increase their knowledge of STH, leads to protective hygiene practices to prevent STH^12,23^. The findings of an intervention study revealed that the implementation of health-education package to prevent worm infections in China demonstrated an effective reduction of STH infections in the participating schoolchildren^24^.

On the other hand, our study indicated that the knowledge and practice scores between infected and uninfected adults did not differ significantly except for *Trichuris* infection. Adults infected with *Trichuris* tended to have a significantly lower practice score than those without the infection. It is unclear why the knowledge and practice scores were consistently higher in the uninfected children than the infected ones, but such patterns were not observed in adults. For example, adults infected with *Ascaris* had higher knowledge and behaviour levels than those uninfected adults. However, those adults with *Trichuris* infections tended to have a significantly lower behaviour score than the uninfected ones (64.08 vs. 69.11). As noted by recent studies on examining KAP and parasitic worm infections, acquired knowledge did not necessarily translate into behavioural changes^15,19,25^. For instance, Sady et al.^21^ argued that poverty could have contributed to infected individuals’ delay in seeking treatment even though they showed a higher level of knowledge than those without infections. This was because they did not have enough money to pay the cost of transport and medical services, especially for residents living in rural or remote communities. In such communities, inadequate sanitation and poor environmental conditions (such as a lack of access to clean water) could be the key barriers to community deworming or STH health education programs. In addition, there was also misconception regarding the effect and accessibility of treatment. For example, some study participants considered that the effect of preventative chemotherapy was permanent and repeated treatments were not required^19^; or the treatment was ineffective, inaccessible and not affordable^12,15,18,22^. As a result, STH re-infections could be a common issue among adult residents in rural communities, even though they might have previously been exposed to STH prevention and health education programs and had good knowledge of prevention of STH infections. As Acka et al.^15^ suggested, STH prevention and treatment programs should combine community-based health education campaigns and school-based interventions to enhance and sustain positive sanitation and hygiene practices in rural settings.

The model validation results further confirmed that the quantitative measurement tools developed in this project and subsequent related projects are suitable for assessing knowledge and behaviour associated with STH. The validation findings in this study demonstrated great model performances in predicting knowledge and behavior scores, when applying the scales to the study population. Using the basic demographic variables of the study sample, we can correctly predict respondents' knowledge and practice scores to a great extent (85–88% agreement between observed and predictive scores). The first validated model showed that education level is a significant predictor for knowledge scores. This finding is consistent with previous KAP studies that education was significantly associated with KAP on STH or schistosomiasis infections^8,21,22^. However, these studies did not establish association based on overall knowledge or practice scores. They examined the associations based on individual questions included in the scales. Nevertheless, this finding highlights the importance of health education programs in communities. Our finding also indicated that existing relevant studies often displayed frequency distributions of individual KAP items under each sub-category of knowledge or practice questionnaires^8,15,16,21^. To our knowledge, there are no validated quantitative instrument available to measure the levels of STH-related knowledge and behaviour. There is also a lack of standardized scales or tools to determine adequate levels of STH-related knowledge and practices. In order to inform future STH prevention (in relation to specific forms of STH infections), further research efforts can be made to determine the optimal cut-off points for adequate/inadequate levels of knowledge and practices with regard to STH to identify the target population for further population-based health education programs.

In conclusion, the results of this study have demonstrated promising validation outcomes for the knowledge and practice scales included in the BALatrine questionnaire with the study population (adults and children). They can be used to aid population health education programs and to increase sustainability of mass deworming and environmental improvements. The findings further confirmed the importance of integrating school-based (targeting children) and community-based (targeting adults and households) health education or STH prevention programs to maximize the effect of STH prevention and intervention programs.

## Supplementary Information

Supplementary Information 1.

Supplementary Information 2.

Supplementary Information 3.

## Data Availability

Data collected as part of this study are not publicly available but can be shared upon reasonable request by emailing the corresponding author.
