# Supplementary material for: Model validation for a knowledge and practices survey towards prevention of soil-transmitted helminth infections in rural villages in Indonesia
Source: Sci Rep. 2023 Jan 25;13:1444. doi: 10.1038/s41598-023-27781-3 (PMC9876981; doi:10.1038/s41598-023-27781-3)
Supplement: Supplementary file 3 — Supplementary Information 3. [file 41598_2023_27781_MOESM3_ESM.docx]

**Supplementary Table 1**

(A) Comparison of descriptive statistics between observed and predicted knowledge scores (%)

|  | Observed knowledge %  (N=6466) | Predicted knowledge %  (N=6238) |
| --- | --- | --- |
| Mean (SD) | 54.73 (13.33) | 54.49 (9.58) |
| Median | 60.00 | 56.92 |
| Percentiles |  |  |
| 25 | 52.00 | 50.62 |
| 50 | 60.00 | 56.92 |
| 75  100 | 64.00  88.00 | 61.09  75.26 |

(B) Comparison of descriptive statistics between observed and predicted behaviour scores (%)

|  | Observed knowledge %  N=6466 | Predicted knowledge %  N=6238 |
| --- | --- | --- |
| Mean (SD) | 67.62 (14.97) | 67.47 (11.62) |
| Median | 65.85 | 66.98 |
| Percentiles |  |  |
| 25 | 58.54 | 60.37 |
| 50 | 65.85 | 66.98 |
| 75  100 | 78.05  100.0 | 76.27  97.78 |


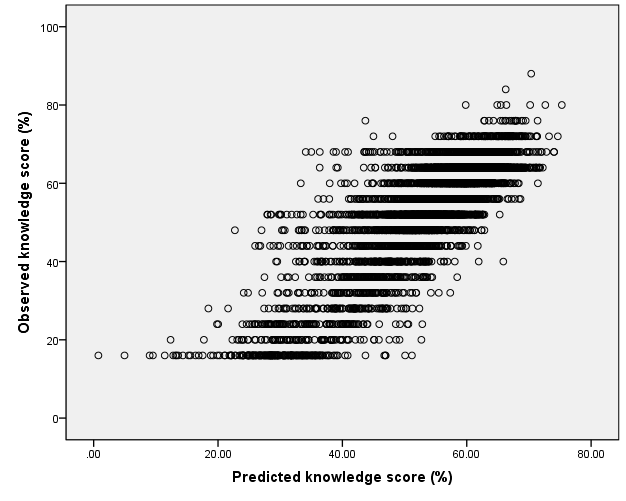


rho= 0.847

P<0.001

**Supplementary Figure 1** Correlation between observed and predicted knowledge scores (%)


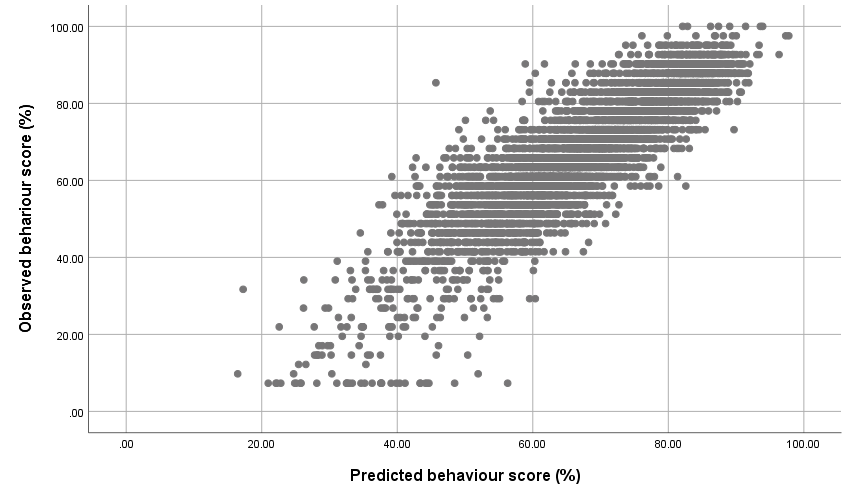


rho= 0.883

P<0.001

**Supplementary Figure 2** Correlation between observed and predicted behaviour scores (%)
